# Adapting to vision changes: nighttime driving experiences of older adults

**DOI:** 10.3389/fragi.2025.1563917

**Published:** 2025-08-06

**Authors:** Christopher R.M. Rundus, Dominique Rose, Fangda Zhang, Sean Brown, Jingzhen Yang

**Affiliations:** ^1^ Center for Injury Research and Policy at the Abigail Wexner Research Institute, Nationwide Children’s Hospital, Columbus, OH, United States; ^2^ Department of Sociology, Columbus State University, Columbus, OH, United States; ^3^ Department of Pediatrics, The Ohio State University, Columbus, OH, Ohio State

**Keywords:** nighttime driving, older drivers, road safety, qualitative, driving independence

## Abstract

**Introduction:**

This qualitative study explored older adults’ perspectives and experiences of nighttime driving, focusing on age-related vision changes.

**Methods:**

We conducted virtual, semi-structured interviews with active drivers aged 60 or older from central Ohio. The interview guide encompassed four key domains: personal and driving history, nighttime driving practices/abilities/anxieties, the role of driving, and policy opinions. Interviews were transcribed verbatim and analyzed using thematic analysis with ATLAS.ti™.

**Results:**

Eleven participants (eight males; ages 64–77) completed interviews. Four major themes emerged: (1) age-related vision and driving changes, (2) nighttime driving patterns, (3) importance of driving, and (4) nighttime driving safety recommendations. Each theme included two to three subthemes, focusing on perceived impact of vision changes on nighttime driving ability and potential adaptive strategies to address these changes.

**Discussion:**

Our findings contribute to existing literature and may inform programs and technological advancements to enhance nighttime driving safety for this vulnerable population.

## 1 Introduction

Every day in the United States, the safety of older drivers emerges as a critical concern. Statistics reveal that more than 20 drivers aged 65 and older are killed, and nearly 540 are injured in crashes daily (Centers for Disease Control and Prevention, 2024). This demographic also faces an elevated risk of fatal nighttime collisions, ranking second only to drivers under 25 in terms of nighttime fatalities per mile driven (Massie et al., 1995; Cicchino and McCartt, 2014; Mortimer and Fell, 1989). Age-related changes in vision, both anatomical and functional, significantly contribute to this heightened risk, resulting in a two to four times greater likelihood of fatal crashes at night compared to daytime (Cicchino and McCartt, 2014; Mortimer and Fell, 1989; Kimlin et al., 2017). Another major obstacle for older drivers navigating nighttime conditions is oncoming headlight glare (Ortiz-Peregrina et al., 2020; Van Den Berg et al., 2009). However, current clinical vision assessments typically prioritize visual acuity under daytime photopic lighting, without including mesopic (low light) and glare conditions that are critical during nighttime driving (Wood and Chaparro, 2011; Wood, 2020; Wood and Owens, 2005).

Despite driving less overall, and especially at nighttime (Molnar et al., 2015; Molnar et al., 2018), older adults continue to face a disproportionately high risk of fatal nighttime crashes per mile driven (Massie et al., 1995; Cicchino and McCartt, 2014; Mortimer and Fell, 1989). Age-related visual impairments, such as reduced contrast sensitivity and increased sensitivity to glare, likely contribute to this heightened risk (Kimlin et al., 2017; Ortiz-Peregrina et al., 2020). Recent surveys underscore older drivers’ awareness of these risks, with over one-third expressing concerns about their driving abilities, particularly at night (Allen et al., 2019). While prior research has highlighted both the functional limitations and perceived risks associated with aging, there remains a need to better understand how older adults navigate these challenges. Given the substantial increase in the number of older drivers—48 million licensed in 2020, a 68% rise since 2000 (Beck et al., 2022; Owsley et al., 2020)—understanding the factors influencing nighttime driving becomes imperative for maintaining both individual driving privileges and public road safety. Insights into older drivers’ perspectives on nighttime driving could inform targeted interventions aimed at improving their safety and mobility in an aging society.

This qualitative study aimed to explore older drivers’ views and experiences regarding influences of age-related vision on their ability of driving at night, and adaptive strategies they recommended to ensure safe driving at night for drivers 65 or older.

## 2 Methods

### 2.1 Study design and participants

This study used a qualitative research approach and researchers conducted semi-structured interviews with 11 active drivers aged 60 and older from central Ohio to explore their perceptions and experiences regarding their nighttime driving. To be eligible, a participant needed to have a valid driver’s license, engage in driving activities at least once per week, and consent to be interviewed. All participants had previously completed our larger visual function and driving simulated assessment study. Detailed descriptions about study participants and recruitment strategies are reported elsewhere (Yang et al., 2024). Our exclusion criteria included individuals who drove less than once per week, relied on visual aid devices beyond habitual correction (e.g., bioptic lens), or had medical conditions (e.g., epilepsy, vertigo) that prevented them from driving regularly). Individuals who were non-English speakers or with a history of severe motion sickness were also excluded.

### 2.2 Study procedures

Upon completion of the larger study, eligible participants were recruited for our interview study. If they expressed interest, a virtual interview was scheduled. An experienced qualitative researcher (S.B.), trained on the study protocol, conducted the 11 interviews via Microsoft Teams^®^ between March and August 2022. Only the participant and researcher were present for the interview. Each interview, which lasted between 45 min and 1 h, was audio-recorded. Participants provided verbal consent immediately prior to the interview. Following each interview, the audio recordings were sent to a third party for verbatim transcription. The transcribed interviews were then uploaded to ATLAS.ti™ (Version 22) (ATLAS.ti Scientific Software Development GmbH, 2023). A qualitative data management software application, to facilitate the coding and analysis process. To protect their identities, all participants were assigned pseudonyms. We reviewed each transcript as it became available, prior to conducting the subsequent interview, to ensure the integrity of the transcriptions and make necessary modifications to the interview guide. This study received ethical approval from the Institutional Review Boards at the authors’ institutions (STUDY00000461). This report follows Consolidated criteria for reporting qualitative research (COREQ) reporting guidelines for qualitative interview studies (Tong et al., 2007).

### 2.3 Interview guide

We developed a qualitative research question to explore the lived experiences of older adults with nighttime driving amid declining visual function. A literature review informed initial domains and constructs of the semi-structured interview guide (Kimlin et al., 2017; Wood, 2020; Gruber et al., 2013; Andersen, 2012), which were then refined with input from experts in the field, including a public health researcher specializing in traffic safety and a vision researcher with extensive experience in aging. The interview guide covered four key domains, each directly related to nighttime driving decisions: (Centers for Disease Control and Prevention, 2024): personal and driving history (Massie et al., 1995), nighttime driving practices, abilities, and anxieties (Cicchino and McCartt, 2014), the role of driving in daily life, and (Mortimer and Fell, 1989) opinions on driving licensure policies. The questions probed into participants’ experiences with nighttime driving, factors that affect their comfort levels, strategies they employ to manage nighttime driving anxiety, and their observations on how their driving habits have evolved over time. The guide also included questions that solicited participants’ views on potential technological solutions and policies that could enhance nighttime driving safety for older adults, as well as their suggestions for improving awareness and resources related to nighttime driving. The interview guide was pilot tested before being finalized.

### 2.4 Data analysis

The thematic analysis approach (Braun and Clarke, 2006), was employed to explore the perceptions and experiences of active drivers aged 60 and older regarding their driving particularly nighttime driving. A preliminary codebook was established based on the interview guide and an initial review of the transcripts, defining broad categories of findings to facilitate the coding of interview responses. This preliminary codebook was applied to three transcripts to validate the coding definitions and identify additional codes emerging from the data before refining and finalizing the codebook. Intercoder reliability was established, and frequent meetings were held between research team members to ensure agreement and resolve discrepancies. Once the codebook was finalized, two coders (S.B and D.R) then independently undertook line-by-line coding of the 11 interview transcripts using ATLAS.ti™ software (ATLAS.ti Scientific Software Development GmbH, 2023). They met weekly to resolve discrepancies and ensure coding consistency. After coding was completed, the research team including the coders met to discuss any remaining disagreements and identified the statements or codes mentioned by the participants and grouped them into larger categories based on their similarities (Lochmiller, 2021; Chinh et al., 2019; Ide and Beddoe, 2024). These categories were subsequently synthesized into the most significant themes of the study findings. The coded data based on the agreed-upon themes were then synthesized into meaningful themes and subthemes (Lochmiller, 2021). This process was iterated until no new themes were identified. The data were analyzed at both the semantic (surface) and latent (interpretive) levels (Lochmiller, 2021). The semantic analysis focused on participants’ explicit statements, illustrated through direct quotes in the results section and Table 1. The latent analysis, which explored underlying meanings, is presented in the discussion section.

**TABLE 1:** Themes, subthemes, and sample quotes.

Themes	Subtheme	Quotes
Theme A. Age related vision and driving changes		
	A.1. Age related vision change	“I’ve noticed as I’ve gotten older, driving at night is a little harder, just the vision aspect. Not necessarily the lights, but the lack of, of light. Let’s put it- … that way” (Participant, 001).
		“I think my reactions are still … I mean, as you get older, your reaction time and your muscle tone is not the same, but I think … I would consider it above average at this point” (Participant, 008).
	A.2. Driving habit changes due to age	“Yeah, I used to go on long trips, and no problem. You do not grab all those miles that I drove if you did not like it, you would not do that. No, it was fine until lately. It’s just more difficult. It seems busier, and lots of traffic up here, where I live now. There used to be in a lot less traffic out in the country, where I lived. And, I’m just older” (Participant, 009)
		“At this time in retirement, both in our, my husband and I are retired. I do not drive much cause he does most of the driving at this point.” (Participant, 005)
		“I do not go out quite socially, because being out of the house 5 days a week … you do not quite recover. When you’re 30, going out after work is a little bit different than when you’re 50 or 60” (Participant, 009)
Theme B. Nighttime driving patterns		
	B.1. Level of comfort with nighttime driving	“I had cataract surgery a few years ago and that decreased my vision, my nighttime vision, which bothers me because I had really good nighttime vision before the cataract surgery, but I do not mind driving, at least since I’m not driving out of town. I do not know how it would be driving out of town, but I know [urban city]. And so it does not bother me to drive at night” (Participant, 011)
		“I do not have anxiety about driving at night … uh, maybe my night vision driving is not as good as it was 40 years ago, you know? And I am aware of that … so when I get into heavy traffic at night, I am more cautious than I, than I would’ve been otherwise … “ (Participant, 001)
		“[Nighttime driving] Is just not as enjoyable. I have to concentrate more. (Participant, 005)
	B.2. Perceived ability of nighttime driving	“I’m still confident enough in my driving to do it. I’m not to a point where I would say, “No, I’m not ever going to drive at night” (Participant, 002)
		“When I say that I believe I’m a good driver at night, it also means that I think the most important thing, for drivers to drive within their abilities, you know, not to outdrive, what they see or, what they, can make out as far as their vision in the road and things” (Participant, 001)
		“Yeah, I think it’s fine. If I were going to rate it on a scale of one to 10, I would say a nine at least” (Participant, 008)
		“At least adequate. It’s a task, I work on it. If I have to drive at night, I work at it. I have done that. I took a trip, had to come back from the airport … [and] I took a taxi that time … I just do not have any reason to go out at night now, really, so I do not” (Participant, 009)
		“Yes. I am comfortable driving at night. I do not have a problem with it. I just do not do it a lot right now. Again, it’s the early morning times when it’s still before sunrise that is my night driving” (Participant, 009)
	B.3. Adaptive strategies for nighttime driving	“Well, and it really depends on what we’re doing, if we’re traveling somewhere. I’ll drive 4 or 5 h nighttime on a trip. But we do plan our trips to leave early in the morning. So it usually starts off in darkness, before we leave, and then, daybreak comes and we drive all day and then we’ll maybe drive a few hours into the evening until we get tired … Probably [during] the average week about 6 or 7 h” (Participant, 001)
		“No, not really. It does not matter. If I want to go someplace and it’s dark, I do not care, I just go” (Participant, 008)
Theme C. Importance of driving		
	C.1. Importance of maintaining a driver’s license	“Well, I think it would make me anxious only in the sense that I would have to figure out who and how to drive for me at night. Again, I’ve never done an Uber. I’ve never done any kind of ride sharing. So, I’d have to figure that out. I’m not a good [night time driver], “Ask my neighbor,” or ask a friend, “Hey, come and get me.” So, again, that would raise some anxiety about my ability to ask for a favor” (Participant, 009)
		“It does mean independence. If needed. That’s the big thing” (Participant, 005)
	C.2. Perception of nighttime driving restrictions	“Well, I think that would probably be a good idea. When it got applied to me, if it wound up imposing a restriction, I would not be happy. But if it would have a net that caught a lot of people who should not be driving at night, I guess that would be for the good” (Participant, 006)
		“I really think that would be an important thing to have” (Participant, 001)
		“It would take away a lot of people’s driver’s license. I’m sure. I got friends that drive to and from church and to and from the grocery store and that’s all they drive, and they know that route like the back of their hand. And I think they’re totally adequate to do that. And I think that would take that away from them. I think it would affect a lot of people … “ (Participant 011).
Theme D: Participant recommendations		
	D.1. Technological solutions	“So those are some of the things you asked about, improvements that could be made to help the older folks. I think all of those things come into play. Better roads, the autonomous vehicles, better marking of the roads, better lights that do not glare into your eyes at night in the rural areas, all those things I think come into play” (Participant, 008)
		“If more roads had those (reflectors as lane markers), that would be very helpful” (Participant, 005)
		“It seems like the new light bulbs that they put in [vehicles], some of ‘em are blue colored you know, some of them are really harsh and … seem to bother [my vision] more” (Participant, 002)
	D.2. Nighttime vision test	“I do not know how they would do it. That would be a difficult thing to, I think, a difficult thing to simulate … “ (Participant, 008)
		“I think maybe the eye exam part of it maybe should be every 2 years. You know, I say that because of how much my eyes have changed in the last 2 years” (Participant, 001)
	D.3. Public transportation and rideshare programs	“Sure, I’m all for public transportation. More public transportation of any kind, I’m for all those kind of things. To get people out of cars and into public transportation, especially in the city, that’s a big deal” (Participant 009)
		“I think certainly the travesty is … Midwestern cities lack adequate public transportation. So I’m one of the people who rail all the time about the fact that there’s no light rail or subway … So I would want to see more of a commitment to public transportation options beyond the bus system, which is good, but limited. I would have to walk about a mile to catch a bus that would[n’t] get me anywhere useful, you know? (Participant, 010)

### 2.5 Ethics approval

The study received ethical approval from the Institutional Review Boards at the authors’ institutions (IRB # STUDY00000461).

## 3 Results

Of the 11 (Molnar et al., 2015) individuals interviewed, three were female (27%). All the participants were non-Hispanic White, with their age ranging from 64 to 77 years (mean = 69.45, SD = 4.80). Four major themes were identified from the interviews: (1) age-related vision and driving changes, (2) nighttime driving patterns, (3) importance of driving, and (4) participant recommendations (Table 1).

### 3.1 Age related vision and driving changes

Age related vision and driving changes was operationalized as current adjustments in participants’ driving habits due to their advanced age and their age-related vision change. Two subthemes were identified, including age-related vision changes and age-related driving habit changes.

#### 3.1.1 Age related vision change

Several participants discussed changes to their vision and highlighted the challenges they were facing in their daily life, with one participant explaining, “[My vision] is on the decline. I noticed when I read street signs and license plates now, I can read ‘em, but they're like, they're fuzzy or double … I mean, my vision's not very good” (Participant, 001). Another interviewee stated, “The last time I had my eyes checked, which was about, six months ago, the doctor told me that I have the beginning of cataracts” (Participant 002). Some participants reported the impact of their slower visual adjustment to light changes due to advanced age: “It takes me a little longer to adjust to the changes in light. If I'm in bright light and then suddenly go to dark, it's going to take me a little bit longer than it used to maybe to adjust, not much” (Participant 010).

#### 3.1.2 Driving habit changes due to age

Participants expressed that as they have grown older, they became more cautious drivers. For instance, one respondent stated, “[I’m] driving a little slower, being more cautious of people around me. Ice I avoid. Unless it's like an emergency, just do not go” (Participant, 005). Another mentioned, “I’ve slowed down a lot in my old age” (Participant, 009). Participants also mentioned a decrease in their driving activities, with one participant stating, “About 20 years ago, I retired from work. And before then, I was probably doing more driving in general …“ (Participant, 003). Another commented, “I used to go on long trips, and no problem. You do not grab all those miles that I drove if you did not like it, you would not do that. No, it was fine until lately. It's just more difficult” (Participant 009).

### 3.1 Nighttime driving patterns

Nighttime Driving Patterns was operationalized as the level of comfort participants felt with driving at night, how they perceived their own nighttime driving ability, and strategies utilized to ease nighttime driving. Three subthemes emerged from this theme: level of comfort with nighttime driving, perceived ability of nighttime driving, and adaptive strategies implemented for nighttime driving.

#### 3.2.1 Level of comfort with nighttime driving

Most participants expressed comfort when initially asked about nighttime driving. However, many reported discomfort when encountering glare from oncoming headlights, making nighttime driving very difficult: “The glare is really bad. I usually do not drive at night unless I absolutely have to” (Participant 002). Participant 001 shared this sentiment saying, “one of the biggest problems from older drivers is the glare from headlights, cars coming the other way, or the streetlights.” Another participant referenced LED lights saying, “Now it is the way they make lights now, not only headlights, but other kind of lights too, it is this big glare and it does make it difficult, especially on the ones who have those blue headlights” (Participant 011).

Participants expressed difficulty and discomfort when driving at night when adverse conditions were present: “depending on the conditions. As I say, when it is raining, it is awful” (Participant 002). Participant 009 felt similarly about feeling anxiety when driving in the rain sharing, “A little bit, yeah. Especially in the rain, same thing.”

#### 3.2.2 Perceived ability of nighttime driving

All participants indicated the deterioration of their visual capabilities as they aged, which profoundly affected their nighttime driving. One respondent described their current nighttime driving ability with that of 20 years ago and said, “I’m sure as a younger person, 20 years ago, I had better eyesight than I do now. Better reaction time” (Participant 011). Participant 010 stated “I just know what my limits are. If there really was no street lighting, no nothing. It was really dark, say it was a moonless night. I would probably curtail my speed a little bit.” Many participants shared that the increased visual difficulties made their nighttime driving more difficult, requiring greater concentration: “It is just not as enjoyable. I have to concentrate more” (Participant 005). This difficulty was reflected through many interviewees with another saying, “I’ve noticed as I’ve gotten older, driving at night is a little harder, just the vision aspect. Not necessarily the lights, but the lack of light.” (Participant 005).

In contrast, participants who underwent cataract surgery felt their nighttime vision improved after the surgery, which alleviated their previous apprehensions. For instance, one participant noted the stark difference post-surgery, saying, “Driving at night, I have no problem now. Before I had the cataract surgery, it was scary.” (Participant 008). This participant continued saying, “Prior to that though, most of the time, most of my night driving was very local and on roads that I knew.”

#### 3.2.3 Adaptive strategies for nighttime driving

Participants shared adaptive strategies they employed to manage nighttime driving. One of the common strategies was adjusting their driving times to avoid or minimize nighttime driving altogether. Participant 009 shared that they limit driving at night, “As little as I can. I do not have any reason to go out at night most the time, so I do not.” Many participants planned their trips meticulously to ensure most of their driving occurred during daylight hours: “I arrange longer trips so that I can complete most of the driving in daylight, and if it works out that I have to drive some after dark, I can do that” (Participant 004).

Another adaptive strategy participants shared involved relying on familiar routes when driving at night. One interviewee described this approach, stating, “As a practical matter, most of the driving that I do at night is in the Columbus area, so I’m familiar with where I’m going. If I were going on a trip and I’d be out of town, maybe I’d prefer getting to my destination before it gets much past dinner time” (Participant 006).

Additionally, seeking assistance from others was a common way that was shared to avoid nighttime driving. Some even chose to avoid nighttime driving altogether by finding someone else to drive them to ensure their safety and comfort: “I’ll find somebody to take me. I will not choose to drive at night if I do not have to” (Participant 002). Relying on others to drive however, was not something participants always wanted to do. Participant 011 expressed how relying on others can be burdensome for them, “I could call on a friend here or there, but you can wear friends out real fast.”

### 3.3 Importance of driving

The importance of driving was operationalized as the significance of maintaining a driver’s license and how losing their license would impact their lives. Two subthemes were identified from this theme: importance of maintaining a driver’s license, and the perception of nighttime driving restrictions.

#### 3.3.1 Importance of maintaining a driver’s license

Participants emphasized the significance of retaining their driver’s license and considered emotional, practical, and social implications of maintaining this privilege. Participants emphasized that maintaining a driver’s license allowed them to be mobile: “I always had cars and been able to drive. So, that would be a pretty drastic change if I could not drive” (Participant 003). Getting around meant more than going on drives for enjoyment. Participants shared how driving allows them to meet basic life needs, getting food, shopping for necessities, and visiting people: “Oh, it means everything, it means my food. It means my entertainment. It means shopping for necessities. Means visiting friends and family. I know I dread the day when I can’t drive anymore. Hopefully I’ll be dead or in a nursing home by then and will not have to worry about driving” (Participant 011). This melancholy outlook on a life without driving was emphasized by Participant 003 who shared, “I would feel lost if I could not drive.”

#### 3.3.2 Perception of nighttime driving restrictions

Participants were introduced to the idea of putting a restriction on driving at night for drivers with age-related decline in vision to protect drivers as well as other road users. Participants largely expressed acceptance for this restriction but shared it would not be okay to take away their license altogether: “I think it would be (good) if it was not a limiting factor to get a driver’s license” (Participant 002). Participants also noted that it would be good to keep people who cannot meet driving requirements off the roads, “It would probably be a good thing. I do not want blind people driving at night” (Participant 009).

Participants drew parallels between a nighttime restriction and other restrictions that already exist (glasses, etc.). One respondent stated, “You always need to be wearing your glasses when you’re driving. If it were something like that with the night driving, so that it did not keep you from having a driver’s license to drive during the day” (Participant 002). Participants shared how the restriction should not be selective based on age, but all ages should test for nighttime vision: “Now if the nighttime vision part of it was selective based on age, I might question that and say, ‘Okay.’ But if you have it, why not give it to everybody because while it is rare that young people, middle aged people will have nighttime vision problems, it is not beyond the realm of possibility” (Participant 004).

### 3.4 Participant recommendations

The final theme, participant recommendations was operationalized as the suggestions and solutions provided by study participants to improve nighttime driving safety including alternatives to nighttime driving. Three subthemes were identified from this theme: technological solutions, mandatory nighttime vision test, and public transportation and rideshare programs.

#### 3.4.1 Technological solutions

Participants noted that advanced technologies such as Advanced Driver Assistance Systems (ADAS) and self-driving cars could significantly enhance nighttime driving ability and safety of seniors. One respondent expressed enthusiasm about the potential of self-driving cars, “Well, I kind of like the developments going on with the self-driving cars. I think that would be really useful to a lot of people. I think that a computer can probably drive a car better than most people” (Participant 003). Another participant highlighted specific ADAS features that could compensate for slower reflexes, “I might be more inclined to think about getting a car model after these cars run their course. That would have things like lane assist, for example, or some kind of braking assist just to account for the difference in my reflexes” (Participant 010).

Regarding a common issue of glare from incoming vehicles’ headlights or streetlights, one participant offered a suggestion for a technological solution to cut out some of the glare, “One of the biggest problems for older drivers is the glare from headlights, cars coming the other way, or the streetlights. If there could be some kind of, I do not know if ambient lighting inside the car could offset some of that” (Participant 001).

Participants also emphasized the importance of road infrastructure improvements to enhance nighttime driving. One suggestion was the increased use of reflectors as lane markers to improve visibility, “If more roads had those (reflectors as lane markers), that would be very helpful” (Participant 005).

#### 3.4.2 Nighttime vision test

Participants expressed a desire to continue driving as long as possible, but they also acknowledged the safety implications of driving with poor nighttime vision. They recognized the importance of balancing safety with independence. One participant reflected on the idea of nighttime driving tests, “It is not a bad idea. Right off I’m like, ‘I do not want them telling me that.’ But as we know, seniors start losing night vision so for safety reasons it would be a good idea to just test that” (Participant 005).

Participants also highlighted the significance of protecting themselves as well as other road users, even if it meant restricting nighttime driving: “The night vision testing would help things. You’ve got to protect people from themselves sometimes, as difficult it is. If that happened to me, it would be really difficult, but I just would not drive at night” (Participant 009).

#### 3.4.3 Public transportation and rideshare programs

Participants emphasized the value of having transportation alternatives beyond personal vehicles. Participants expressed the need for more accessible and cost-free public transportation options. They highlighted how financial constraints can limit their ability to use public transit, which is vital for those on fixed incomes: “Having more public (transit) and without cost ‘cause let’s face it, when you retire and you’re on a fixed income, sometimes money is an issue” (Participant 005). Another interviewee discussed the need for trains saying, “So I’m one of the people who rail all the time about the fact that there’s no light rail or subway” (Participant 010).

Participants noted the lack of reliability of current transportation systems, especially for those who depend on them for essential appointments. They stressed the need for improvements in scheduling and service reliability. Participant 011 described the challenges faced by their friends, “I have friends who are disabled now at this age and they depend on the COTA transport bus, but it is so unreliable and you might have to wait for 2 h. You have to schedule their appointment for 2 h ahead of their doctor’s appointment. And then they may get you there on time or late. And then you may have to sit and wait for two more hours” (Participant 011). Participants also discussed the limited accessibility of public transportation, particularly in rural areas: “mass transit in the United States is not the answer, because in my area, that’s not quite available.”

## 4 Discussion

This study explored the perspectives and experiences of eleven older drivers (aged 64–77 years) on nighttime driving, focusing on the impact of age-related vision changes. Participants shared their views and practices on the adjustments in driving habits due to age-related vision decline, challenges and strategies for managing nighttime driving, importance of maintaining a driver’s license, and suggestions for improving nighttime driving safety through technological and policy interventions. This study contributes rich qualitative insight into how these challenges are internalized and addressed in everyday life. Given that one in every four licensed drivers will be 65 or older by 2050 (Beck et al., 2022), the results of this study have significant implications for the nighttime driving safety of near 50 million licensed drivers in this age group. Most participants reported that age-related vision changes have significantly impacted their ability to drive at night, especially when encountering glare. The finding aligns with previous studies, which consistently identify glare as a major obstacle for older drivers during nighttime driving (Kimlin et al., 2017; Ortiz-Peregrina et al., 2020; Van Den Berg et al., 2009; Wood, 2020; Friedland et al., 2017). To enhance safety, participants shared various adaptive strategies, such as avoiding nighttime driving when possible, steering clear of traffic, and avoiding specific road types. This self-regulation by driving less at night or avoiding difficult situations in response to declining abilities mirrors findings in existing literature (Molnar et al., 2015; Beck et al., 2022; Liang et al., 2022). Further research is needed to identify the most effective practices older drivers can use to improve road safety and reduce crashes and fatalities at night so that healthcare providers working with older adults can be informed when offering recommendations to older drivers who face challenges with nighttime driving.

Existing literature highlights strong correlations between car ownership, driving, independence, and life satisfaction in older adults (Chihuri et al., 2016; Choi et al., 2014; Fonda et al., 2001; Marottoli et al., 1997; Ragland et al., 2005). Participants in this study echoed this sentiment, expressing considerable concern about the possibility of losing their ability to drive. While most participants wanted to retain their licenses, they acknowledged the importance of road safety and were open to the idea of nighttime driving restrictions for those experiencing significant difficulties. Such restrictions would allow older drivers to preserve their driving privileges and independence while ensuring safety during the nighttime, when they are most vulnerable. Healthcare providers working with older adults and their families could also offer personalized recommendations on nighttime driving based on their vision and other health conditions, helping to balance independence and safety without completely revoking driving licenses.

Through their own experiences, participants suggested numerous approaches to enhance driving ability and safety among older adults, particularly when driving at night. Many emphasized the potential value of self-driving cars and advanced driver assistance systems (ADAS), technologies increasingly integrated into modern vehicles with features like lane-keeping assistance, adaptive headlights, and collision warnings, all designed to help drivers make quick decisions in critical moments (Mueller and Cicchino, 2022; Östling et al., 2019; Sezgin and Lin, 2019). When properly utilized, these tools may reduce crash risk and support continued mobility for older adults, especially those with age-related visual impairments (Wood et al., 2024; Greenwood et al., 2022). Healthcare providers may offer personalized guidance to help older adults and their families make informed decisions about nighttime driving based on their individual functional needs and preferences. This may include discussing the potential benefits of vehicles equipped with advanced driver assistance systems (ADAS). Additionally, promoting accessible alternatives such as rideshare services or public transit can support mobility and reduce reliance on nighttime driving, a recommendation that is consistent with existing literature emphasizing the importance of transportation alternatives for aging populations (Jones et al., 2018).

Another potential solution to tackle older drivers’ nighttime driving challenges involves policy change. Implementing nighttime vision tests for older people could increase awareness of visual limitations and support safer driving decisions. The Bureau of Motor Vehicles (BMV) may consider implementing such tests specifically for nighttime vision and applying license restrictions for those who do not meet the required standards. This could help ensure that older drivers, especially those with vision impairments, are aware of whether their vision limitations pose a safety risk when driving at night.

## 5 Limitations

One limitation of this study is that the sample included only active drivers from Ohio, which may limit the generalizability of the findings to older drivers in other regions or those who are no longer driving. Further, the nature of semi-structured interviews is such that researchers can steer conversations in general directions that may not always correspond with the spontaneous reflections of respondents. Additionally, despite intercoder reliability and the attempt to achieve consensus in any analytical disputes, the possibility of biased analysis or interpretation remains. Finally, due to the small sample size, potential sex-based differences in nighttime driving experiences were not explored; future research with a larger sample size should explore these differences in more detail.

## 6 Conclusion

This study highlights the challenges older drivers face due to age-related vision changes, particularly at night and when encountering glare from oncoming headlights. Our findings contribute to the existing body of literature and emphasize the need for further research to explore various approaches and strategies to help older adults manage these challenges effectively. This includes technological solutions to enhance nighttime safety and alternative transportation options, such as door-to-door pick-up and drop-off services, to reduce nighttime driving exposures. Given that the older adult population is expected to nearly double by 2050, future research should evaluate the effectiveness of these strategies in reducing nighttime crashes and fatalities. This will provide valuable pieces of evidence to inform guidelines and policies, ensuring older drivers stay safe while maintaining their independence and access to essential services and social participation.

## 7 Reflexivity

We engaged in reflexive practices throughout the study to acknowledge how our identities, experiences, and context shaped the research (Olmos-Vega et al., 2023). Researchers’ backgrounds ranged from Public Health to Engineering. Team discussions regularly addressed power dynamics, particularly in participant–researcher interactions, and informed adjustments to our approach. Methodologically, we aligned with a constructivist stance, viewing knowledge as co-constructed, and adapted our analytic strategies through collaborative coding. We also considered how broader social and institutional contexts, such as concussion recovery norms, influenced participants’ narratives and our interpretations.

## Data Availability

The raw data supporting the conclusions of this article will be made available by the authors, without undue reservation.

## Abbreviations

ADAS, Advanced Driver Assistance Systems; BMV, Bureau of Motor Vehicles; COREQ, Consolidated Criteria for Reporting Qualitative Research.
